# Faba Bean: Unlocking nutritional potential and agricultural sustainability

**DOI:** 10.1016/j.crfs.2025.101136

**Published:** 2025-07-03

**Authors:** Madhvi Singh, Maria Balota, Haibo Huang, Sean O'Keefe, Renata Carneiro

**Affiliations:** a Virginia Polytechnic Institute and State University, Department of Food Science and Technology, Virginia, USA; b Virginia Polytechnic Institute and State University, Department of Plant Pathology, Physiology and Weed Science, Virginia, USA

**Keywords:** Faba beans, Broad beans, Vicine, Convicine, Antinutritional factors

## Abstract

Faba bean (*Vicia faba* L.) has been identified as a versatile specialty crop for North America due to its rich nutritional profile, ability to thrive in diverse climates, and economic potential. Although friendly to most diets, faba bean consumption is challenged by the presence of vicine and convicine—antinutritional compounds that trigger favism in individuals with glucose-6-phosphate dehydrogenase deficiency. This review delves into the genetic, molecular, and biochemical dimensions of vicine and convicine accumulation in faba beans, pinpointing knowledge gaps and unearthing historical and lesser-known insights about these compounds.

This comprehensive review synthesizes and discusses recent efforts and challenges in enhancing nutrition of faba beans to promote its production and consumption in North America. We spotlight the strides made in breeding low-vicine and low-convicine varieties and critically assess attempts aimed at mitigating these favism-inducing factor**s.** The development of low-vicine and low-convicine faba bean lines represents a significant advancement in crop breeding, addressing safety concerns for individuals with G6PD deficiency. By utilizing marker-assisted breeding techniques, researchers are effectively reducing vicine and convicine levels, even as the complete biosynthetic pathways of these compounds remain unresolved. Current research efforts are steadily progressing toward cultivars with minimal or no vicine and convicine, enhancing the safety and nutritional profile of faba beans. These breakthroughs hold the potential to transform faba beans into a more sustainable, inclusive, and widely consumed food source, expanding their utility in both human diets and agricultural systems.

## Highlights

• Faba beans are rich in protein, essential amino acids, fiber, and bioactives, contributing to sustainable nutrition.
• Antinutritional factors reduce nutrient bioavailability and pose risks to individuals with G6PD deficiency.
• Identification of the VC1 gene enables marker-assisted selection of low-vicine, low-convicine cultivars.
• Fermentation, enzymatic hydrolysis, and thermal treatments effectively lower vicine and convicine content.
• CRISPR/Cas-based editing and agronomic optimization enhance the sustainability of faba bean cultivation.

## Graphical abstract

**Figure:**
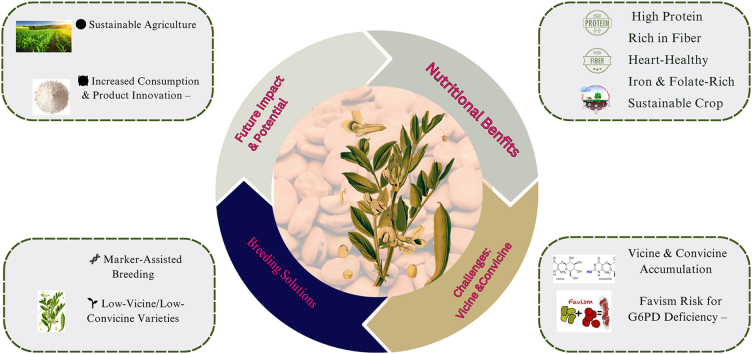

## 1 Introduction

As the global demand for sustainable and nutritious food sources grows, faba bean (*Vicia faba* L.) emerges as a crop with immense potential to address pressing challenges in agriculture and nutrition. Known for its exceptional nutritional profile and ecological benefits(xiu Xiao et al., 2021), faba bean is among the earliest domesticated crops, with a history dating back to the late 10th millennium B.P. in northwest Syria (Tanno and Willcox, 2006). Today, it is widely cultivated in countries such as China, Ethiopia, the United Kingdom, and Canada, covering over 6.4 million acres globally. In North America, while Canada has established significant production, the crop remains underexplored in other regions, such as the United States. The U.S. accounts for only 3 % of global green faba bean production, with approximately 40,000 acres harvested annually between 2020 and 2022 (World Food and Agriculture, 2024) Despite currently being underutilized, faba bean holds tremendous economic potential for the United States (FAOSTAT 2021, 2021)With 62 % of U.S. households—approximately 79 million—purchasing plant-based protein products, the plant-based food market grew from $4.8 billion in 2018 to $7.4 billion in 2021 (FAOSTAT, 2021, 2021). This trend reflects the rising consumer demand for sustainable and nutritious diets, aligning with the global protein alternatives market, which is projected to grow from $114.4 billion in 2024 to $126.3 billion by 2028 (Global Legumes Market Worth USD). With its high protein content—nearly double that of most pulses—and adaptability to diverse growing conditions, faba bean could become a pioneer crop for protein-based products in the U.S. market.

Faba bean is a nutritional powerhouse, rich in protein, dietary fiber, iron, zinc, and essential vitamins such as folate, riboflavin, and thiamine (Tiwari and Singh, 2023). These attributes, combined with its low-fat content and bioactive compounds, contribute to various health benefits, including reduced risks of colorectal cancer and cardiovascular diseases, improved gut health, and lower blood cholesterol levels (Aune et al., 2011), (Clemente and Olias, 2017), (Sharma et al., 2011)The versatility of faba beans extends to their culinary applications, where they are featured in Mediterranean and Asian dishes such as *falafel*, *medammis* (stewed beans), and *bissara* (bean paste). Faba bean flour has gained recognition for its role in gluten-free products like bread and pasta, with studies showing that incorporating faba bean flour can enhance protein content and improve functionality (Sozer et al., 2019), (Giménez et al., 2012).

In addition to its nutritional value, faba bean plays a critical role in promoting agricultural sustainability. As a leguminous crop, it enriches soil fertility by fixing atmospheric nitrogen and reducing the need for synthetic fertilizers (Jensen et al., 2010), (Hardarson et al., 1991). When integrated into no-till systems, faba bean can lower greenhouse gas emissions from cultivated land (Šarauskis et al., 2020). These ecological benefits, combined with its high yield potential, make faba bean a promising candidate for addressing sustainability challenges in agriculture.

Despite these advantages, the widespread adoption of faba beans is limited by the presence of antinutritional factors (ANFs), which interfere with nutrient absorption and utilization. Among ANFs, vicine and convicine are pyrimidine glycosides found in faba beans that hold particular significance due to their implications for food safety and nutritional quality. These compounds reduce protein digestibility, impair mineral bioavailability, and, upon hydrolysis, produce aglycones associated with favism in individuals with glucose-6-phosphate dehydrogenase (G6PD) deficiency (Khazaei et al., 2019).

Advances in plant breeding have enabled the development of low-vicine and low-convicine cultivars, thereby mitigating these concerns (Bjerg et al., 1984), (Kumar et al., 2015). Processing methods such as soaking, fermentation, and enzymatic treatment have further shown promise in lowering vicine and convicine levels, enhancing the nutritional quality of faba beans for both human and animal consumption (Singh and Kaur Arora, 2023). By integrating these approaches into breeding programs and production practices, the U.S. can harness faba beans as a key player in the shift toward sustainable, plant-based protein systems.

This comprehensive review explores the chemical characteristics of vicine and convicine, as well as their biosynthesis, nutritional implications, and health effects. Additionally, it discusses methods for quantifying these compounds and current genetic and breeding approaches to reduce their content in faba beans. The goal is to provide a roadmap for mitigating these antinutritional factors, unlocking the full potential of faba beans as a sustainable and nutritious crop for North America.

## 2 Nutritional value of faba beans

**Fig. 1:**
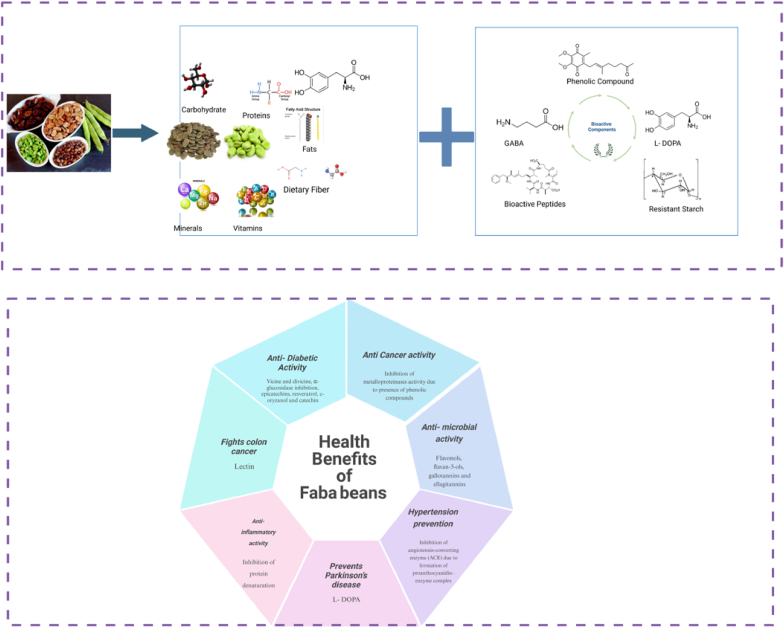
Schematic illustrating faba beans' nutritional profile, bioactive compounds, and their health benefits.

The rich nutritional profile of faba beans makes this legume an attractive resource to improve nutrition in North America. Faba beans contain high levels of lysine-rich protein, complex carbohydrates, dietary fiber, and bioactive compounds such as phenols, γ-aminobutyric acid (GABA) and antioxidants (Khazaei et al., 2019), (Liu et al., 2022).They are also a valuable source of essential minerals like iron, zinc, potassium, and magnesium (Haciseferoǧullari et al., 2003), (Rahate et al., 2021). Fig. 1 represents the nutritional composition, bioactive components, and potential health benefits of faba beans (Martineau-Côté et al., 2022a).

### 2.1 Protein

The protein content in faba beans ranges from 20 % to 41 %, depending on the variety and processing methods (Yang et al., 2018a). The primary proteins in faba beans are globulins (60 %), albumins (20 %), glutelins (15 %), and prolamins (8 %) (Yang et al., 2018b). Faba beans are a rich source of lysine and an exceptional source of plant-based protein, particularly valuable in regions where access to animal protein may be limited. Table 1 provides a detailed breakdown of the protein composition and an amino acid profile of faba beans in comparison to other legumes. This table highlights the high levels of essential amino acids such as lysine, which contribute to faba beans' role as a versatile protein source in plant-based diets.

**Table 1:** Nutrient profile: Average amino acid (g/kg) and protein contents (%) in Twelve legume crops.

Legumes	Ala	Arg	Asp	cys	Glu	Gly	His	ILe	Leu	Lys	Met	Phe	Pro	Ser	Thr	Trp	Tyr	Val	Protein
Soybean	0.75	1.23	1.87	0.34	3.43	0.81	0.44	0.83	1.46	1	0.25	0.95	0.94	1.04	0.69	0.24	0.57	0.82	36–39
Pea	1.4	0.6	8.9	0.2	12.8	0.6	0.4	0.6	1	1	0.18	1	0.8	0.8	0.8	–	0.4	1	22–24
Faba Bean	9.39	21.2	22.1	3.22	32.9	8.82	5.96	8.49	14.5	13	2.18	8.54	7	8.15	7.08	–	6.74	9.87	27–32
Chickpea	4.97	8.3	11	0.6	17.3	3.7	3	4.8	8.7	7.2	1.1	5.5	3.8	3.7	3.1	0.9	2.8	4.6	22
Pigeon Pea	3.79	5.86	11.56	1.19	9.23	3.07	3.66	3.47	6.78	7.79	1.19	6.15	3.17	3.59	3.12	–	2.63	5.85	22.3
Green gram	4	6.96	1.8	0.7	20.5	8.96	2.5	5.8	7.9	3.9	1.8	5.6	3.17	7.45	4.5	1.12	7.5	5.6	20.9–31.2
Black gram	2.23	3.4	3.4	0.5	1.5	2.2	1.5	2.7	4.3	3.5	0.8	3.1	6.1	2.2	0.8	0.5	1.6	2.9	26
Lentil	3.65	3.7	–	0.1	–	–	2.09	3.6	6.6	–	0.6	4.7	–	–	3.3	–	–	4.02	24–26
Cowpea	3.65	6.4	–	0.1	–	3	2.4	2.8	5.7	–	1.2	4.2	4.6	4.2	2.9	–	5	3.3	22–24
Kidney beans	1.02	1.5	2.94	0.26	3.7	0.9	0.7	1.1	1.9	1.7	0.4	1.3	1.0	1.32	1.02	0.3	0.7	1.3	22.9

*ala - A* alanine, *arg - R* arginine, *asp - D* aspartic acid, *cys - C* cysteine, *glu - E* glutamic acid, *gly - G* glycine, *his - H* histidine, *ile - I* isoleucine, *leu - L* leucine, *lys - K* lysine, *met - M* methionine, *phe - F* phenylalanine, *pro - P* proline, *ser - S* serine, *thr - T* threonine, *trp - W* tryptophan, *tyr - Y* tyrosine, *val - V* valine.

### 2.2 Carbohydrates

Faba beans contain 44**-**47 % carbohydrates, with starch comprising 41–58 %, which provides a steady source of energy (USDA, 2021). The main soluble sugars include raffinose, stachyose, and verbascose, belonging to the raffinose family of oligosaccharides (RFOs). While these oligosaccharides offer health benefits as prebiotics, supporting gut bacteria and promoting mineral absorption, they can also cause gastrointestinal discomfort and gas production due to fermentation by gut microorganisms (Mayer Labba et al., 2021). The concentration of these RFOs varies significantly across cultivars, necessitating careful management through breeding and processing to optimize their benefits while minimizing digestive issues.

### 2.3 Dietary fiber

A rich source of dietary fibre, including both soluble and insoluble types, ranging from 8 % in whole beans to over 82 % in seed coat (Singh et al., 2014), (Çalışkantürk Karataş et al., 2017). Dietary fibre is essential for maintaining digestive health, reducing cholesterol, and regulating blood sugar levels.

### 2.4 Bioactive compounds

Various bioactive compounds are found in faba beans, including antioxidants, phenolic compounds, flavonoids, and Gamma-Aminobutyric acid (GABA). These compounds offer numerous health benefits, such as reducing oxidative stress, lowering the risk of chronic diseases like cardiovascular diseases and diabetes, and improving overall metabolic health (Khazaei et al., 2019), (Liu et al., 2022).

### 2.5 Minerals

Mature seeds contain significant amounts of iron (6.7 mg/100 g), magnesium (192 mg/100 g), potassium (1062 mg/100 g), and phosphorus (421 mg/100 g) (USDA FoodData Central). These minerals are vital for various physiological functions, including oxygen transport, muscle contraction, and bone health.

### 2.6 Vitamins

In addition to minerals, faba beans are a good source of vitamins, including folate (423 μg/100 g), vitamin C (1.4 mg/100 g), and niacin (2.832 mg/100 g) (USDA FoodData Central). Folate is essential for DNA synthesis and repair, vitamin C is an antioxidant that supports the immune system, and niacin is essential for energy metabolism.

## 3 Anti-nutritional factors and health implications of faba beans

The presence of vicine and convicine, along with other antinutritional compounds such as tannins, phytates, and trypsin inhibitors, negatively impacts digestibility of proteins and absorption of minerals. These compounds bind with proteins and minerals, forming less digestible and absorbable complexes, which in turn reduce the bioavailability of essential nutrients such as iron and zinc (Gu et al., 2020). Table 2 summarizes the key antinutritional factors in faba beans and their effects. Among these factors, vicine and convicine are unique due to their specific and severe effects on individuals with G6PD deficiency (Chen et al., 2017). While other common antinutritional factors include lectins, tannins, and phytates, which impair nutrient absorption, reduce protein digestibility, and bind essential minerals such as iron and zinc, the impact of vicine and convicine is more critical (Chen et al., 2017). These compounds can lead to potentially fatal anemia in susceptible individuals, highlighting their importance in dietary considerations and food processing (Chen et al., 2017).

**Table 2:** Anti-nutritional factors in faba beans (Salvador-Reyes et al., 2023).

Anti-Nutrient	Chemical nature	Health Impact	Potential Benefits	Amount
Vicine and Convicine	Pyrimidine glucosides	- Cause favism, a hemolytic anemia, in G6PD-deficient individuals. - Decrease feeding efficiency in animals.	- Act as natural fungicides and insecticides for plants. - Potential resistance to pests.	4.96 ± 1.97 mg/g 1.98 ± 0.84 mg/g
Protease inhibitor	Proteins that inhibit protease enzymes	- Decrease protein digestibility by inhibiting enzymes like trypsin and chymotrypsin. - Lead to non-competitive inhibition of digestive enzymes.	- Play a role in plant defense mechanisms. - Control protein hydrolysis during germination.	2.24–2.77 Trypsin inhibitors units (TIU)/mg
Trypsin Inhibitors	Proteins with reactive amino acids like lysine	- Inhibit trypsin and chymotrypsin, reducing protein absorption.	- Contribute to plant resistance against pests.	8.01 ± 6.04 Trypsin inhibitors units (TIU)/mg
Tannins	Polyphenols (condensed and hydrolyzable)	- Bind to proteins, decreasing their digestibility. - Reduce bioavailability of minerals such as iron.	- Prevent cardiovascular diseases and act as anticancer agents.	10–96.50 mg/100g
Phytic Acid	Hexaphosphoric acid ester	- Binds minerals like calcium, magnesium, iron, reducing their bioavailability. - Inhibits enzymes like amylase, pepsin, and trypsin.	- Reduces starch absorption and digestion. - Acts as an antioxidant and has hypolipidemic effects.	21.70 ± 11.05 mg/g
Lectins	Carbohydrate-binding proteins	- Bind to glycoproteins, reducing nutrient absorption. - Can cause food allergies and digestive issues.	- Potential anticancer effects. - Beneficial effects in small doses.	1.37 ± 1.06 Hemagglutinin units (HU)/mg

### 3.1 Vicine and convicine: key anti-nutritional factors

Vicine and convicine are pyrimidine glycosides predominantly found in the faba bean seeds (*Vicia faba* L.) (Smith et al., 2020). Structurally, these compounds feature a pyrimidine ring attached to a glucose molecule (Jones, 2018). Vicine is identified explicitly as 2,6-diamino-4,5-dihydroxy-pyrimidine-5-(β-D-glucopyranoside), while convicine is 2,4-diamino-6-hydroxy-5-(β-D-glucopyranoside)-pyrimidine, differing by an additional hydroxy group (Jones, 2018). These glycosides are primarily synthesized within the developing pods of the plant, reaching their highest concentrations in the cotyledons of both fresh and dry seeds (Lee. and Nguyen, 2019).

Upon ingestion, vicine and convicine undergo enzymatic hydrolysis by β-glucosidase in the gut, releasing the aglycones divicine and isouramil (Davis and Kim, 2021). These aglycones induce oxidative stress in red blood cells by producing reactive oxygen species (ROS), which are especially harmful to individuals with glucose-6-phosphate dehydrogenase (G6PD) deficiency (Lee, 2019). This deficiency impairs the ability of cells to regenerate reduced glutathione (GSH), a vital antioxidant that protects cells from oxidative damage. Glutathione peroxidase plays a key role in detoxifying harmful peroxides by using GSH, thereby reducing oxidative stress in red blood cells. In individuals with G6PD deficiency, the limited capacity to regenerate GSH weakens the body's antioxidant defenses, leading to increased oxidative stress, cellular damage, and ultimately hemolysis. This oxidative damage can trigger acute hemolytic anemia, known as favism, particularly after consuming oxidant-rich foods like faba beans (Lee, 2019).

Interestingly, the reduced ability to regenerate GSH in individuals with G6PD deficiency is closely linked to malaria. The *Plasmodium parasite*, which causes malaria, relies on a reduced (low-oxidative) environment to thrive within red blood cells. In G6PD-deficient individuals, the reduced capacity to regenerate GSH leads to a more oxidized intracellular state, which creates an unfavorable environment for the parasite's survival. Thus, G6PD deficiency offers a natural protective advantage against malaria by making red blood cells less hospitable to the parasite. This evolutionary trade-off providing some resistance to malaria while increasing the risk of hemolysis under oxidative stress explains the persistence and prevalence of G6PD deficiency in malaria-endemic regions (Cappellini and Fiorelli, 2008).

### 3.2 Favism: A hemolytic syndrome

Favism is triggered by compounds in faba beans, specifically vicine and convicine, which are metabolized into their toxic aglycones divicine and isouramil (Cardador-Martínez et al., 2012), (Getachew et al., 2018). These aglycones generate ROS, such as hydrogen peroxide, through redox cycling mechanisms. In individuals with G6PD deficiency, the reduced ability to regenerate GSH exacerbates oxidative damage, resulting in hemoglobin denaturation, membrane lipid peroxidation, and premature destruction of RBCs in the spleen(Beretta et al., 2023).

Clinically, favism manifests with symptoms such as jaundice, pallor, dark urine, tachycardia, abdominal pain, and fatigue. The severity of these symptoms depends on the amount and ripeness of faba beans consumed, the individual's age, and the specific G6PD mutation. Variants such as G6PD Med and G6PD Cairo are associated with more severe outcomes compared to G6PD A**-** (Reading et al., 2016), (Diez de Fuentes, 2016). Laboratory findings typically include anemia, elevated bilirubin levels, and the presence of Heinz bodies, which are aggregates of denatured hemoglobin in RBCs (Beretta et al., 2023), (Sköld et al., 2017).

**Fig. 2:**
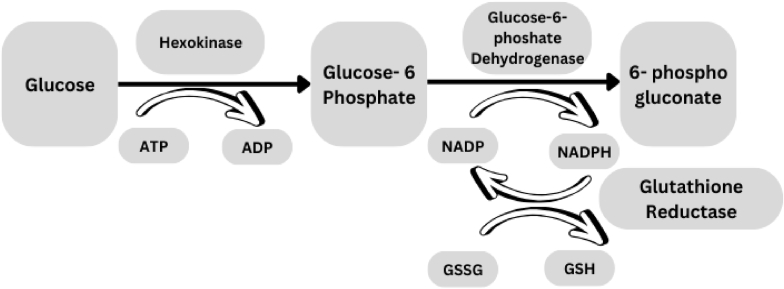
The impact of Glucose-6-Phosphate Dehydrogenase (G6PD) Enzyme Deficiency on the Pentose Phosphate Pathway: Blocking the Initial Step Crucial for Ribose 5-phosphate synthesizing.

Source: [46]

The underlying mechanism of RBC susceptibility lies in the disruption of the pentose phosphate pathway. This pathway is essential for producing NADPH, which is required to maintain GSH in RBCs. In G6PD-deficient individuals, impaired NADPH production limits the detoxification of ROS, leading to oxidative stress (Sintes et al.). Fig. 2 illustrates this process and the critical role of G6PD in maintaining RBC integrity.

### 3.3 Broader health risks of vicine and convicine

In addition to hemolysis, the compounds in faba beans pose other health risks:

• **Neurological Effects**: Vicine has been shown to inhibit adenosine kinase (ADK) activity, leading to increased adenosine accumulation in the central nervous system. This accumulation has been linked to neurological disorders such as epilepsy and seizures in susceptible individuals (Paramashivam et al., 2021).
• **Toxicity of Aglycones**: Although processing methods can reduce vicine and convicine levels, the presence of residual aglycones (divicine and isouramil) after processing remains a concern. These compounds continue to pose risks due to their oxidative potential, which may not be fully neutralized by standard treatments (Pulkkinen, 2019).
• **Exacerbating Genetic Conditions**: Genetic factors beyond G6PD deficiency, such as certain acid phosphatase locus 1 (ACP1) phenotypes with low enzymatic activity, can further increase susceptibility to oxidative damage in RBCs (Polzonetti et al., 2011).

Reducing vicine and convicine levels extends beyond human health, impacting livestock health and growth rates when faba beans are used as animal feed (Singh et al., 2018). High levels of these compounds in feed can lead to reduced animal productivity, underscoring the need for low-vicine and low-convicine varieties in agriculture. Additionally, cultivating faba beans with reduced antinutritional factors can enhance sustainable agricultural systems by improving crop rotation and nitrogen fixation, contributing to environmental sustainability (Singh et al., 2018).

### 3.4 Biosynthesis and distribution

The biosynthesis of vicine and convicine in faba beans follows an unconventional pathway, originating from purine metabolism rather than pyrimidine metabolism as previously assumed. These pyrimidine glucosides are derived from guanosine triphosphate (GTP), a purine nucleotide (Björnsdotter et al., 2021). The key enzyme involved in their biosynthesis is VC1, which exhibits GTP cyclohydrolase II activity, catalyzing a crucial step from GTP. Feeding experiments with labeled ^13^C_10_, ^15^N_5_-GTP confirmed that GTP is a precursor for both compounds in faba beans(Björnsdotter et al., 2021). The VC1 gene was identified through gene-to-metabolite correlation analysis across various faba bean tissues, encoding an isoform of 3,4-dihydroxy-2-butanone-4-phosphate synthase/GTP cyclohydrolase II, typically involved in riboflavin biosynthesis (Ramsay and Griffiths, 1996). A mutation in the VC1 gene, specifically an inactivating AT dinucleotide insertion, is linked to reduced levels of vicine and convicine in specific faba bean cultivars. VC1 expression strongly correlates with vicine content across tissues and co-locates with the significant quantitative trait locus (QTL) for vicine and convicine content (Ramsay and Griffiths, 1996), (Pitz et al., 1980).

The concentration of vicine and convicine is largely governed by genetic factors, particularly the presence of the recessive vc-gene, which is associated with the white hilum trait (Khamassi et al., 2013), (Martineau-Côté et al., 2022b). This gene is the primary determinant of low vicine and convicine levels, with genetic markers now available to distinguish between high (VC) and low (vc-) genotypes, facilitating breeding efforts (Martineau-Côté et al., 2022b). Varietal differences also contribute to the variability in vicine and convicine content among different faba bean germplasm and varieties (Khamassi et al., 2013), (Martineau-Côté et al., 2022b), (Sergeant et al., 2024). In standard high VC varieties, vicine levels typically range from 3.8 to 13.6 mg/g, while convicine levels are 1.5–5.7 mg/g. In contrast, low (vc-) varieties exhibit vicine levels of 0.13–0.73 mg/g and convincing levels of 0.009–0.037 mg/g, a substantial reduction (Martineau-Côté et al., 2022b).

The developmental stage of the faba bean influences vicine and convicine levels, with higher concentrations found in immature seeds than mature seeds (Khazaei et al., 2019; Ali and Shakir, 2023). These levels decrease during seed maturation, likely due to the dilution effect from accumulating storage compounds (Ali and Shakir, 2023). Environmental and processing factors also impact the levels of these glucosides. Enzymatic treatments with β-glucosidase can significantly reduce vicine and convicine content, but this process generates toxic aglycones, such as divicine and isouramil, which necessitate additional processing steps to ensure their complete removal and prevent potential health risks. Factors such as soaking temperature, pH, time, and cooking processes further enhance their elimination.

Although geographic and environmental influences on vicine and convicine distribution are less directly studied, their role can be inferred. The primary determinant is genetic, specifically the presence or absence of the vc-gene, which likely overrides environmental effects. While environmental factors such as temperature, moisture, and soil fertility could influence the biosynthesis and accumulation of these compounds, genetic determinants are the significant drivers. Thus, the geographic distribution of vicine and convicine levels largely depends on the prevalence of high vs. low vicine/convicine varieties cultivated in different regions, driven by breeding objectives rather than environmental adaptation (Ramsay and Griffiths, 1996), (Pitz et al., 1980).

Understanding these biosynthetic pathways and their genetic regulation has laid the foundation for more targeted crop improvement. Recent genomic studies have significantly advanced the understanding of faba bean biology and breeding potential. A high-quality genome assembly of a short-wing petal faba bean genotype (VF8137), characterized by low outcrossing rates, was recently reported by (Liu et al., 2025a) who conducted genome-wide analyses across 558 accessions. Their work identified key genomic regions and candidate genes associated with floral traits, yield, and environmental adaptation, providing critical insights for marker-assisted breeding. Similarly, a reference-grade faba bean genome (∼13 Gb) was produced by (Liu et al., 2025b), demonstrating extensive gene family expansions and structural variation. These genomic resources represent a significant leap forward, enabling precise identification and manipulation of genes underlying agronomically important traits, including those controlling vicine and convicine content. Together, these advances position faba bean breeding to benefit from genomics-assisted strategies aimed at enhancing both nutritional quality and safety.

### 3.5 Interactions with other nutrients and enzymes

Vicine and convicine can interact with various bioactive compounds and enzymes, complicating their nutritional impact and reducing the bioavailability of key nutrients present in faba beans. Vicine and convicine can bind to proteins, forming less digestible complexes. This interaction reduces the efficiency of protein utilization in the body, a significant concern given the high protein content of faba beans. The binding of these compounds to proteins can decrease overall digestibility, thus impacting the nutritional value of faba beans as a protein source (Yang et al., 2018b).

The binding of vicine and convicine to minerals such as iron and zinc reduces their bioavailability. Iron is crucial for oxygen transport and preventing anemia, while zinc is essential for immune function and wound healing. Additionally, faba beans contain phytate, an antinutritional factor that chelates these essential minerals, further decreasing their bioavailability. The phytate content among different faba bean cultivars varies significantly, ranging from 112 mg/100 g to 1281 mg/100 g, depending on factors such as cultivar, climatic conditions, and soil factors. A high phytate-to-mineral molar ratio, as seen in most cultivars with Phy ratios >15, indicates reduced zinc bioavailability, which can impair immune function and wound healing(Mayer Labba et al., 2021). Reduced absorption of these minerals is particularly concerning for populations that rely heavily on faba beans as a dietary staple, as it may lead to iron and zinc deficiencies (Rahate et al., 2020). Faba beans are rich in bioactive compounds, including antioxidants and vitamins such as polyphenols, flavonoids, vitamin C, and folate. Polyphenols, including flavonoids like catechins and phenolic acids, contribute significantly to reducing inflammation and oxidative stress. However, vicine and convicine undergo hydrolysis to produce divicine and isouramil, which induce oxidative stress. This oxidative stress can degrade these antioxidants, reducing their ability to protect against cellular damage and chronic diseases (Khazaei et al., 2019).

Vitamin C is also affected by the oxidative by-products of vicine and convicine. Vitamin C plays a crucial role in supporting immune health, but its degradation limits its efficacy in combating oxidative damage. Similarly, folate, essential for DNA synthesis and cell division, may have reduced bioavailability in the presence of vicine and convicine, negatively impacting populations that rely heavily on faba beans for their nutritional needs (“USDA FoodData Central).

Vicine and convicine can inhibit specific enzymes involved in nutrient metabolism, thereby reducing the efficiency of nutrient absorption and utilization. For example, they may inhibit glucosidase enzymes, which are crucial for carbohydrate metabolism. This inhibition can limit the breakdown and absorption of carbohydrates, reducing the energy available from faba beans (Chen et al., 2017). Additionally, vicine and convicine may interfere with protease enzymes that are essential for protein digestion, leading to reduced availability of amino acids necessary for growth and tissue repair**.**

In summary, vicine and convicine in faba beans pose significant challenges to their nutritional value. These antinutritional factors reduce protein digestibility and mineral absorption and interact negatively with other nutrients. Understanding and mitigating their effects through genetic, breeding, and processing approaches is crucial to enhancing the nutritional benefits of faba beans and ensuring their safety for consumption (Khazaei et al., 2019), (Sharan et al., 2021).

### 3.6 Mitigation strategies

Efforts to mitigate health risks from faba bean consumption focus on two main approaches: processing methods and breeding low-vicine and low-convicine varieties.

Thermal treatments, such as roasting and boiling, can reduce vicine and convicine levels by up to 61 % ((Cardador-Martínez et al., 2012). However, the resilience of vicine and convicine to thermal degradation complicates their complete *elimination* through conventional cooking methods (Martinez and Rodriguez, 2022). Alternative techniques, such as soaking in acidic solutions, enzymatic degradation, and fermentation, have been shown to effectively reduce their content in faba beans (Martinez and Rodriguez, 2022). For example, fermentation with *Lactobacillus plantarum* can degrade over 90 % of these glycosides, showcasing a biotechnological approach to mitigating their antinutritional effects (Martinez and Rodriguez, 2022). Enzymatic hydrolysis and fermentation with lactic acid bacteria (*Lactobacillus plantarum*) offer more significant reductions, with some studies reporting up to 85 % reduction in vicine and 47 % in convicine during fermentation (Pulkkinen et al., 2019), (Rizzello et al., 2016). However, the effectiveness of these methods in eliminating toxic aglycones remains uncertain.

Advances in plant breeding have identified key genetic loci, such as VC1, associated with vicine and convicine biosynthesis (Björnsdotter et al., 2021). Breeding strategies are pivotal in addressing the risks associated with vicine and convicine. Advanced genetic engineering and marker-assisted selection techniques have enabled the development of faba bean varieties with naturally lower levels of these harmful compounds (Watson et al., 2020). These new varieties offer a safer alternative without compromising the beans' nutritional value, facilitating safer consumption in regions prevalent with G6PD deficiency (Watson et al., 2020). Marker-assisted selection (MAS) has enabled the development of faba bean cultivars with reduced vicine and convicine content, minimizing the risk of favism while preserving the nutritional and agronomic qualities of the crop (Khamassi et al., 2013).

Faba beans offer considerable nutritional benefits but present significant health risks for individuals with G6PD deficiency due to the oxidative stress induced by vicine and convicine. The clinical and systemic impacts of favism highlight the need for targeted interventions. While processing and breeding strategies show promise in mitigating these risks, further research is required to ensure the complete elimination of toxic metabolites.

## 4 Improving faba beans for North America

Faba beans are gaining attention as a sustainable and nutritious crop, making them a promising candidate for addressing North America's growing demand for plant-based protein. Recent breeding efforts have focused on tailoring faba beans to meet the needs of North American consumers by improving resilience to environmental stressors, reducing anti-nutritional factors, and enhancing their overall nutritional profile.

Breeding programs for faba beans aim to achieve several key objectives. First, improving resistance to biotic and abiotic stresses is a priority, given the crops’ susceptibility to frost, drought, heat, and waterlogging, particularly during critical growth stages like flowering and pod filling (Maqbool et al., 2010), (Pampana et al., 2016). Additionally, breeding efforts focus on reducing anti-nutritional compounds like vicine and convicine (v-c), which can cause favism in G6PD-deficient individuals. The development of low-tannin phenotypes is another goal, as seed coat tannins limit the use of faba beans in food and feed applications (Webb et al., 2016), (Zanotto et al., 2020). Enhancing agronomic traits like yield, adaptability, and disease resistance while maintaining consumer-preferred qualities such as improved nutritional value is essential for meeting market demands (Khazaei et al., 2021).

Several challenges hinder the improvement of faba beans for North America. Key hurdles include the biosynthetic pathway of v-c remains incompletely understood, limiting the ability to fully eliminate these compounds (Khazaei et al., 2019). Environmental conditions also significantly influence v-c levels, resulting in variability across genotypes and regions (Maalouf et al., 2019). Furthermore, low-v-c genotypes sometimes fail to meet agronomic and market expectations, creating trade-offs between desirable traits (Khazaei et al., 2019). Faba beans are also affected by biotic stressors like chocolate spot (caused by *Botrytis fabae*), Ascochyta blight (*Ascochyta fabae*), and the black bean aphid (*Aphis fabae*), which can reduce yields by up to 60 % under high disease pressure (Frenda et al., 2013), (Hansen et al., 2008). Seed coat tannins further limit their use in food and feed, requiring breeding for low-tannin genotypes (Webb et al., 2016).

Accurate quantification of vicine and convicine (v-c) levels poses a significant challenge to breeding low-v-c faba bean cultivars. The ability to measure these compounds with precision is essential for identifying low-v-c genotypes, yet existing methods face limitations in terms of sensitivity, efficiency, and scalability. Early methods, such as spectrophotometry and colorimetry, were foundational but lacked the specificity needed to detect low concentrations of v-c (Collier, 1976), (Kim et al., 1982). These methods could only detect concentrations in the range of 2600–7740 mg/kg, making them unsuitable for modern breeding programs focused on low-v-c genotypes.

Advanced methods, including liquid chromatography (LC) with ultraviolet (UV) detection, improved selectivity but struggled to quantify low levels of convicine accurately. Techniques such as hydrophilic interaction liquid chromatography (HILIC) and mass spectrometry (MS) have significantly enhanced sensitivity, allowing for the detection of v-c concentrations as low as 1 mg/kg in low-v-c genotypes. High-field asymmetric waveform ion mobility spectrometry (FAIMS), combined with MS, offers the most sensitive and rapid quantification, detecting concentrations as low as 0.1 mg/kg with an analysis time of less than 1 min per sample. However, the high cost and technical expertise required for these advanced methods present a bottleneck in genetic improvement efforts (Purves, 2018), (Wang, 2015). Table 3 summarizes the analytical techniques for v-c quantification, highlighting their detection ranges, advantages, and limitations.

**Table 3:** Analytical techniques for quantification of vicine and convicine.

Methodology	Advantages	Limitations	Vicine Concentration Range (mg/g)	Convicine Concentration Range (mg/g)	References
Spectrophotometry	Suitable for high concentrations	Lacks specificity and sensitivity for low levels	6.90–7.74	2.60–3.28	(Collier, 1976), (Kim et al., 1982), (Sejr Olsen and Hinge Andersen, 1978), (Sixdenier et al., 1996)
LC with UV Detection	Improved selectivity and robustness	Inaccurate at low concentrations, especially for convicine	0.65–7.59	2.09–3.62	(Pulkkinen et al., 2015), (Quemener, 1988)
HILIC-UV	Enhanced quantification for low v-c seeds	Uncertain long-term stability and reproducibility	3.91–5.8	2.01–3.31	Purves et al. (2018a)
LC-MS/MS (SRM)	High selectivity and sensitivity	Labor intensive, Time-consuming chromatographic process	0.172–5.87	0.014–4.09	(Purves et al., 2017), (Purves et al., 2018b)
FIA-SRM	Rapid analysis (less than 1 min)	Vicine interference with convicine determination	0.69–6.59	0.019–4.68	Purves et al. (2018a)
FIA-FAIMS-SRM	Most efficient and accurate, high-throughput	High initial set-up cost	0.15–6.64	0.009–2.73	(Purves et al., 2017), (Purves et al., 2018b)

HILIC-UV- Hydrophilic Interaction Liquid Chromatography with Ultraviolet detection; FIA-SRM - Flow Injection Analysis with Selected Reaction Monitoring; LC - liquid chromatographic; FIA-FAIMS- SRM – Flow Injection Analysis - high-field asymmetric waveform ion mobility spectrometry - selective reaction monitoring; LC-MS/MS (SRM) - Liquid Chromatography-Tandem Mass Spectrometry - Selected Reaction Monitoring.

Addressing these challenges has paved the way for significant advancements. For instance, the identification of the VC1 gene, responsible for catalyzing a key step in the v-c biosynthetic pathway, provides a foundation for developing v-c-free cultivars using modern breeding technique (Björnsdotter et al., 2021). Marker-assisted selection (MAS) has accelerated progress, with robust, high-throughput markers like KASP now enabling precise selection for low-v-c genotypes (Khazaei et al., 2017). Similarly, genetic discoveries for tannin reduction, such as zt1 and zt2, have led to the development of low-tannin varieties supported by molecular markers (Webb et al., 2016), (Rounsevell et al., 1996).

The adoption of genetic engineering tools like CRISPR/Cas genome editing has opened new possibilities for precise trait modifications. For instance, the successful integration of the *PR10a* gene from potatoes demonstrated enhanced abiotic stress resistance in transgenic faba bean lines (O'Sullivan and Angra, 2016). These advances, coupled with high-density genetic maps and transcriptome datasets, enable breeders to target key traits more effectively (Khazaei et al., 2021)). Additionally, processing innovations such as enzyme treatment and fermentation offer scalable solutions to reduce v-c content, complementing genetic efforts (Pulkkinen, 2019).

Improving faba beans for North American consumers requires addressing significant challenges, including anti-nutritional factors, biotic and abiotic stresses, and market preferences. However, advancements in molecular breeding, genetic engineering, and processing technologies provide promising pathways to overcome these hurdles. By integrating these approaches and engaging consumers with the environmental and nutritional benefits of faba beans, researchers and breeders can ensure their adoption as a sustainable and valuable crop for North America.

## 5 Processing methods to reduce and remove vicine and convicine

Enhancing the applicability and utilization of faba beans as a protein source requires essential processing. Numerous studies have investigated various treatments to reduce or eliminate the antinutritional factors effectively. This section discusses the different treatments applied, their effects on antinutritional factors, and their incorporation into new food products in safe amounts. Various methods have been used to decrease the amount of antinutritional content, including soaking, boiling, pressure cooking, air classification, extrusion, and heat treatment. Additionally, bioprocessing techniques such as fermentation, germination, and enzyme treatments have been utilized ((Alonso et al., 2000), (Jezierny et al., 2010).

Table 4 summarizes the effectiveness of various treatment methods. These treatments highlight their potential for enhancing the safety and nutritional quality of faba beans for incorporation into diverse food products.

**Table 4:** Removal of vicine and convicine from faba beans using various treatments and their effectiveness.

Treatment Method		Type	Vicine Removal (%)	Convicine Removal(%)	Reference
Dry Fractionation by using air classification		protein concentrate	0 (+)	0(+)	(SOSULSKI and McCURDY, 1987), (Coda et al., 2015)
Wet Fractionation by using alkaline extraction (pH 10.5)		protein isolate	99	99	Vioque et al. (2012)
Boiling		flour	30	61	Cardador-Martínez et al. (2012)
Roasting			12	40	
Soaking then Autoclaving			50	50	Jamalian and Ghorbani (2005)
Continuous Soaking	Acid (1 %)	whole beans	100	100	Jamalian and Ghorbani (2005)
	Water	whole beans	100	100	
Germination	After soaking	whole beans	84	100	Jamalian (1999)
	After soaking	whole beans	28	30	
		cotyledons	30–40	50	Goyoaga et al. (2008)
Fermentation	*Lactobacillus plantarum*	suspension	100	75	McKay (1992)
	*Fusarium graminearum*		100	100	
	*Aspergillus oryzae*		100	100	
	*L. plantarum*	sourdough	95	91	Coda et al. (2015)
Food Product	almond powder + lemon juice	fababean paste	90	90	(Coda et al., 2015), (Arbid and Marquardt, 1985)

### 5.1 Dehulling and soaking

Pre-treatment techniques such as soaking and dehulling are routinely employed for faba bean seeds to mitigate antinutrient content and shorten cooking duration. Soaking notably reduces phytic acid levels from 21.1 g/kg to 14.6 g/kg, representing a reduction of approximately 30.8 %, and lowers condensed tannin content from 1.95 g/kg to 1.02 g/kg, which is a reduction of approximately 47.7 % compared to raw seeds. This reduction is due to the leaching of antinutrients into the soaking water (Alonso et al., 2000). demonstrated that soaking faba bean seeds before processing can significantly decrease certain antinutrients. A 4-h soak at room temperature decreased hemagglutination activity, oxalate content, and phytic acid levels (Badjona et al., 2023). Modifying the soaking process with acid or alkali components can cause a 100 % reduction in vicine and convicine levels (van Barneveld, 1999) due to the hydrolysis of these pyrimidine glycosides at different pH levels. Additionally, soaking faba beans at 30 °C for 12 h led to a 14.9 % decrease in α-amylase activity. Lower levels of α-amylase inhibitors in soaked beans were attributed to compound seepage during soaking (Johns and Hertzler, 2021). Steeping faba beans led to notable reductions in trypsin inhibitors, decreasing from 22.6 % to 12.7 %, and chymotrypsin inhibitors, from 17.5 % to 11.4 % (Cheynier, 2005). Hemagglutination activity was also reduced to about 0.6–5.2 % after soaking for 4 h at room temperature (Martini et al., 2021). However, soaking can lead to the partial loss of soluble proteins due to the leaching of antinutrients like phytates and oligosaccharides (Abete et al., 2014).

Dehulling is a mechanical procedure that detaches the hulls from the cotyledons of pulses (Reading et al., 2016). The hulls containing tannins can produce phytochemicals, while the cotyledons serve as a rich plant protein source (Luo and Xie, 2013). The protein content of faba beans increases significantly after dehulling, from 30.1-34.8 % to 60.0–60.9 % (Jakubczyk et al., 2019). This increase occurs because tannins are mainly located in the seed's testa, and dehulling significantly lowers tannin content from 1.95 g/kg to 0.15 g/kg (Kader, 1995). However, dehulling can also elevate trypsin inhibitory activity since trypsin inhibitors are found in the cotyledon (Millar et al., 2019), (Saldanha do Carmo et al., 2020). Dehulling also raises phytic acid levels due to the smaller amounts of phytates in the hulls (Mudryj et al., 2014). This is important for nutritional quality considerations since phytic acid inhibits iron absorption. Additionally, dehulling can improve the palatability and taste of some legumes, which should be investigated for faba beans (Hutchins et al., 2012).

Utilizing soaking and dehulling can effectively reduce antinutrients and enhance the nutritional quality of faba beans. The soaking process, particularly when combined with exogenous compounds like Ca^2+^, Mg^2+^, Na^+^, L-glutamic acid, can direct metabolic pathways to reduce antinutrients further and enhance the nutritional profile. Research on the hydration kinetics of faba beans and the effects of soaking temperature on their nutritional and physicochemical properties is needed. Additionally, exploring technologies such as ultrasound-assisted steeping could reduce soaking times. Utilizing the discarded soaking water, which may contain valuable compounds, is another area for potential research.

### 5.2 Cooking, autoclaving and irradiation

Thermal processing, including cooking, autoclaving, and irradiation, effectively reduces antinutritional factors in faba beans through mechanisms such as thermal degradation, leaching, and structural modifications. These processes involve complex reactions, including deamidation, hydrolysis of peptide bonds, and disruption of disulfide bonds, contributing to the reduction of trypsin inhibitor activity (TIA) and other undesirable compounds (Alonso et al., 2000), (Luo and Xie, 2013).

Cooking at 100 °C for 30 min (bean-to-water ratio of 1:10) reduces TIA by 40 %, while autoclave at 121 °C and 1.6 × 10^6^ Pa for 20 min achieves a 65 % reduction. A combination of microwave heating for 6 min followed by hot air drying at 50 °C for 12 h results in a 55 % reduction in TIA (Luo and Xie, 2013). Autoclaving significantly reduces lectin activity by 75–100 % in green beans and 87–100 % in white beans, while cooking decreases tannin content by 14.9–17.8 % in white beans but does not affect tannin levels in green beans (Luo and Xie, 2013). Additionally, cooking eliminates chymotrypsin inhibitors and reduces hemagglutination activity by 93.8–99.8 % after heating at 95 °C for 1 h (Martini et al., 2021). Pre-soaking and cooking further reduce total oxalate content by 30 %, mitigating the risk of mineral-binding and kidney stone formation associated with oxalates (Martini et al., 2021).

Thermal processing also enhances the nutritional quality of faba beans. A combination of autoclaving, soaking, and dehulling improves in vitro protein digestibility (IVPD), increasing digestibility by 17.4 % in green beans and 11.2 % in white beans (Luo and Xie, 2013). Although phytic acid content may increase by 7.9–10.9 % due to water loss and concentration effects during cooking, reductions in tannins and oxalates improve overall nutritional value. Conventional heating disrupts plant tissue structure, promoting the leaching of phytochemicals, which can result in the loss of antinutritional compounds or nutrients depending on treatment conditions (Cardador-Martínez et al., 2012).

Heat treatment also effectively reduces vicine and convicine levels, highly susceptible to thermal denaturation. Pressure cooking at 122 °C results in a 30 % reduction in vicine and a 61 % reduction in convicine, with variability depending on faba bean variety (Cardador-Martínez et al., 2012). These findings demonstrate the potential of thermal processing to improve the safety, digestibility, and nutritional quality of faba beans, making them more suitable for dietary applications.

### 5.3 Fermentation

Fermentation, involving the biochemical activity of microorganisms, has been widely applied to faba beans to improve their nutritional profile and reduce antinutritional factors. The use of *Lactobacillus plantarum* VTT E 133328 effectively reduced compounds associated with favism and enhanced protein and starch digestibility. This process also increased free amino acids and γ-aminobutyric acid (GABA), a beneficial neurotransmitter (Coda et al., 2015). Processing techniques, including fermentation, led to significant reductions in phytate and tannins, while non-starch polysaccharides (NSP) remained relatively unaffected (Adamidou et al., 2011a). Fermentation reduced vicine and convicine levels by up to 90 %, alongside a 40 % decrease in trypsin inhibitors and condensed tannins (Coda et al., 2015), (Verni et al., 2017).

Conducting fermentation at 30 °C for 48 h lowered the final pH to 4.1, which reduced starch retrogradation and enhanced protein digestibility. This process also increased the levels of GABA and essential amino acids, further improving the nutritional quality of faba beans (Rinne et al., 2020). Additionally, fermentation is pivotal in producing silage, a preserved feed for livestock. Studies on ensiled crimped faba beans have demonstrated that *Lactobacillus plantarum* inoculants enhance lactic acid production and fermentation quality, improving nutritional stability. Formic acid-based additives, on the other hand, minimized fermentation losses and protein degradation (Rinne et al., 2020).

In food applications, fermented faba bean flour exhibits reduced antinutritional factors and improved nutritional properties, making it suitable for products such as baked goods, protein beverages, and high-protein diets (Sozer et al., 2019). Using *Lactobacillus plantarum* as a starter reduced vicine and convicine by 91 % while preserving proteins, essential amino acids, and minerals like iron and zinc (Coda et al., 2015), (Verni et al., 2017). A specific strain, *Lactobacillus plantarum* DPPMAB24W, carrying β-glucosidase, completely degraded vicine and convicine within 48 h (Rizzello et al., 2016).

A broader study on Mediterranean faba bean accessions found fermentation improved protein digestibility to 90 % while effectively reducing antinutritional compounds (Verni et al., 2017). Fractionation techniques, such as air classification and aqueous fractionation, further enhanced the quality of faba bean products. While dry fractionation concentrated antinutritional factors in protein-rich fractions, aqueous fractionation removed over 99 % of favism-causing glycosides and yielded higher protein content, albeit with higher energy requirements (Vioque et al., 2012).

In summary, fermentation not only enhances the nutritional value of faba beans but also offers versatile applications in food and feed industries, promoting better health and sustainability.

### 5.4 Enzyme treatment

Enzymatic treatment is frequently applied due to its advantages, including mild processing conditions, precise reaction control, and low by-product generation. Phytase enzymes originate mainly from two sources: microorganisms (3-phytase) and seeds of higher plants (6-phytase). 3-phytase primarily hydrolyzes phytic acid from the third phosphate group, whereas 6-phytase targets the sixth phosphate group, leading to differences in the specific breakdown pathways of phytic acid. Food-grade phytase is chiefly extracted from fungi of the genus Aspergillus (Karsma, 2015). These enzymes break down phytic acid into inositol and myo-inositol, functioning optimally at a pH of 5.1. In higher plants, phytase becomes active during germination to provide phosphorus for the growing embryo (Oatway et al., 2001). Studies have shown that a 3-h incubation with exogenous phytase can reduce phytate content by approximately 95 % (Luo et al., 2010). Moreover, treating faba bean flour with phytase has enhanced iron absorption in rats (Luo et al., 2010).

### 5.5 Germination and sprouting

Germination is a valuable process for improving the nutritional and functional properties of pulses. It softens cotyledons, reduces cooking time, decreases antinutritional factors, and enhances nutritional quality, aroma, and flavor, thus improving organoleptic properties (Vidal-Valverde et al., 1998), (Roland et al., 2017). This process activates hydrolytic enzymes such as phytase, which are absent in raw seeds and play a crucial role in reducing phytic acid levels (Luo and Xie, 2013), (Dewar et al., 1997).

In faba beans, incorporating sprouts aged 2–6 days into flour and incubating at 30 °C for 30–120 min activated phytase, significantly reducing phytic acid and polyphenol levels. The extent of these reductions depended on sprout age, concentration, and incubation time (Luo and Xie, 2013). Soaking also contributed to polyphenol reduction, primarily through leaching, but caused greater dry matter loss compared to germination. Optimal conditions for reducing phytic acid to 265–66 mg/100 g involved four-day-old sprouts and 120 min of incubation (Luo and Xie, 2013).

A 72-h germination period achieved a 61 % reduction in phytic acid and a 60 % reduction in condensed tannins. Pre-treatment with a 10 % mercuric chloride solution followed by germination in a dark, aerated incubator further reduced antinutritional factors such as α-galactosides and tannins, decreasing RFOs by 45 % and tannins by 60 % (Gilani et al., 2012), (Schwediauer et al., 2018). Notable reductions in condensed tannins occurred within 24–48 h, and proteins were hydrolyzed into peptides and free amino acids, increasing their availability (Vidal-Valverde et al., 1998). However, these benefits must be balanced against nutrient losses due to respiration.

Germination and soaking also improved the in vitro availability of iron and zinc (Luo and Xie, 2013). Short-term germination (24–72 h) enhanced functional properties of faba bean flours, including improved pasting viscosities, emulsion activity, and foaming capacity, likely due to the disruption of protein and fiber matrices surrounding starch granules (Setia et al., 2019). However, longer germination durations (48–72 h) negatively affected pasting viscosities and thermal properties, underscoring the need to optimize germination duration to balance benefits with potential protein degradation.

Overall, germination technology offers significant opportunities for producing pulse flours with enhanced nutritional and functional qualities, making them suitable for various food applications.

### 5.6 Extrusion

Extrusion processing, characterized by high temperature, high pressure, and short exposure time, effectively reduces antinutritional factors while preserving the nutritional integrity of faba beans. This method is widely employed in the feed industry to enhance starch digestibility and reduce the solubility and degradability of plant proteins, thereby improving their nutritional quality (El-Hady and Habiba, 2003).

One of the key benefits of extrusion is the significant reduction in phytic acid levels, which dropped from 21.1 g/kg in raw faba beans to 15.9 g/kg after extrusion—a 25 % decrease. This reduction enhances mineral bioavailability by breaking down inositol hexaphosphate into lower phosphorylated forms, such as tri-, tetra-, and pentaphosphates, which bind less effectively to essential minerals like calcium, iron, and zinc (Alonso et al., 2000). Condensed tannin levels also declined from 1.95 g/kg to 0.89 g/kg, reducing their protein- and carbohydrate-binding properties, which hinder digestion. As a result, in vitro protein digestibility increased from 70.8 % in raw faba beans to 87.1 % after extrusion, underscoring the method's efficacy in improving nutritional value (Alonso et al., 2000).

The reduction of antinutritional factors during extrusion occurs due to thermal degradation, alterations in chemical reactivity, and the formation of insoluble complexes. These changes convert harmful compounds into less active forms, enhancing nutrient bioavailability. For example, the partial breakdown of phytic acid into less mineral-binding forms improves the accessibility of essential minerals (Wang, 2015). Preconditioning, such as wet preconditioning, prior to extrusion further enhances nutritional quality by reducing trypsin inhibitors, which impede protein digestion, thereby improving protein utilization (Adamidou et al., 2011b).

Extrusion processing transforms faba beans into nutritionally robust ingredients with improved digestibility and bioavailability of nutrients. The reduction in antinutritional factors such as phytic acid, condensed tannins, and polyphenols through extrusion enhances their suitability for both human and animal consumption. The process preserves protein content, reduces trypsin inhibitors, and improves mineral absorption, making faba beans a valuable component in food and feed formulations.

Despite its advantages, further research is warranted to explore the interplay between thermal processing, cell wall rigidity, and dietary fiber content to optimize the functional and technological properties of faba beans for diverse food applications.

### 5.7 Wet/dry fractionation

The application of pulses, including faba beans, can be enhanced by fractionation, a process that separates components such as protein, fiber, and starch. Wet fractionation involves aqueous extraction, where water or solvents are used to solubilize and separate different fractions, typically yielding higher protein content but requiring more resources and energy. In contrast, dry fractionation uses mechanical means, like milling and air classification, to separate components, making it a more sustainable process with lower water usage, though the protein content achieved is typically lower compared to wet fractionation ((Augustin and Cole, 2022).

**Fig. 3:**
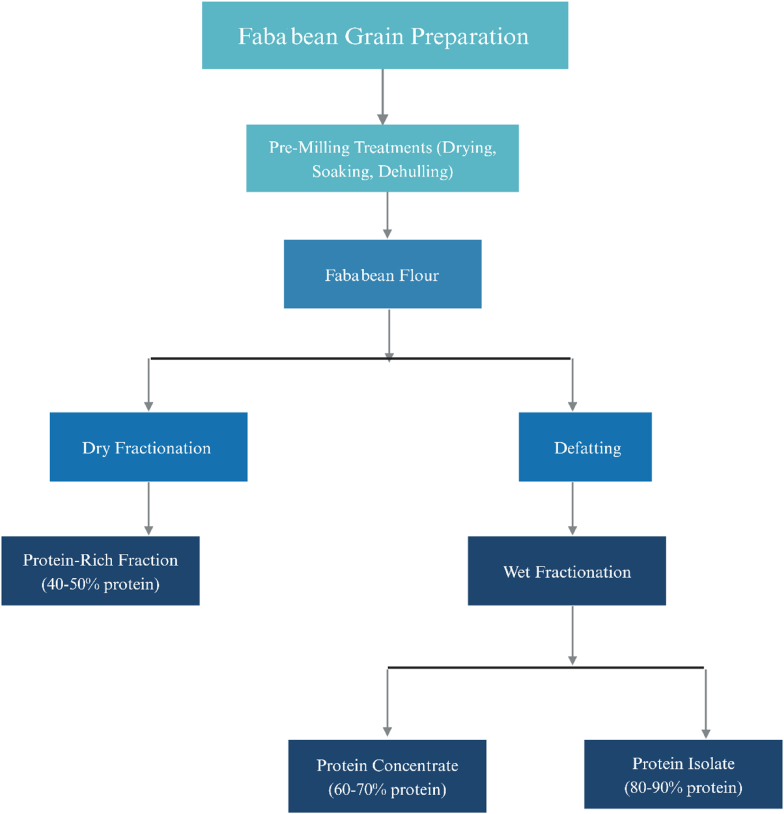
Comprehensive overview of wet and dry fractionation in faba bean processing.

Source: [125]

The fractionation process influences the concentration of vicine and convicine in faba beans. During air classification, these compounds tend to co-segregate with the protein fraction rather than the starch fraction ((Sejr Olsen and Hinge Andersen, 1978), (SOSULSKI and McCURDY, 1987), (Coda et al., 2015). Specifically, protein concentrates containing 63 % protein exhibited vicine and convicine concentrations that were 2.6 and 2.2 times higher, respectively, than those in the flour (SOSULSKI and McCURDY, 1987). Similarly, concentrates with 51 % protein had vicine and convicine levels 1.4 times greater than those in flour (Coda et al., 2015). Starch-rich fractions retained approximately 45 % of the vicine and convicine content compared to flour (Coda et al., 2015). In contrast, protein isolates obtained via alkaline extraction and precipitation at pH4's isoelectric point were nearly devoid of vicine/convicine (SOSULSKI and McCURDY, 1987), (Vioque et al., 2012),with these isolates exhibiting high protein content ranging from 86 % to 92 % (SOSULSKI and McCURDY, 1987), (Vioque et al., 2012). The initial washing of flours with an acetone-water mixture (75:25) facilitated the removal of polyphenols and potentially assisted in eliminating vicine and convicine. Fig. 3 shows the Comprehensive Overview of Wet and Dry Fractionation in Faba Bean Processing.

## 6 Future perspectives and research directions

Faba beans (*Vicia faba*) present significant potential as a sustainable and nutritious food source; however, the presence of vicine and convicine poses challenges due to their antinutritional effects, particularly for individuals with G6PD deficiency who are susceptible to favism. Research into advanced breeding techniques like CRISPR/Cas genome editing offers promising solutions by enabling precise genetic modifications to reduce vicine and convicine concentrations while maintaining or enhancing the nutritional profiles of faba beans. Unlike traditional breeding, CRISPR/Cas allows for faster development of safer, low-vicine, and low-convicine varieties.

Innovations in food processing technologies also hold significant promise. Techniques such as enzymatic hydrolysis, fermentation, and thermal treatments like roasting and autoclaving have demonstrated their ability to significantly reduce vicine and convicine levels. These processes could be optimized for industrial-scale production, enhancing the safety and marketability of faba bean-based products.

Understanding the detailed biosynthetic pathways of vicine and convicine is another critical research area. Further exploration into molecular and enzymatic processes, coupled with tools like single nucleotide polymorphism (SNP) markers and QTL mapping, can accelerate breeding programs by enabling precise selection for low-vicine and low-convicine traits.

Addressing genotype-environment interactions is vital, as these significantly influence vicine and convicine levels in faba beans. Environmental factors like soil composition, climate, and agricultural practices affect glycoside concentrations, making it crucial to select genotypes optimized for specific environments. By minimizing these levels, faba bean crops can consistently meet safety and nutritional standards.

From an environmental perspective, faba beans offer immense benefits as a plant-based protein source. Transitioning to faba bean-inclusive diets could reduce greenhouse gas emissions and resource use compared to animal-based proteins, while their nitrogen-fixing ability supports sustainable agriculture. For example, increasing the production of high-protein crops like faba beans could reduce greenhouse gas emissions by 10–16 % annually and significantly lower land usage.

Advancements in analytical techniques, including high-performance liquid chromatography (HPLC) and mass spectrometry (MS), are crucial for quantifying vicine and convicine concentrations. These tools enable researchers to assess the effectiveness of breeding and processing interventions, ensuring that faba bean products meet safety standards. Moreover, enhancing consumer acceptance through functional applications in plant-based meat alternatives and dairy-free products can broaden their appeal, contributing to healthier and more sustainable diets.

## 7 Conclusion

Advancing the safety, nutritional quality, and consumer acceptance of faba beans require a multidisciplinary approach that integrates genetic, biochemical, and technological innovations. Reducing vicine and convicine levels through advanced breeding techniques like CRISPR/Cas and optimized food processing methods is essential to ensure the safety and nutritional value of faba beans. Additionally, leveraging their environmental benefits, such as nitrogen fixation and reduced greenhouse gas emissions, positions faba beans as a sustainable alternative to animal-based proteins. By addressing consumer demand for plant-based proteins and exploring innovative food applications, such as gluten-free products and plant-based meat alternatives, faba beans can emerge as a cornerstone of sustainable, plant-based diets in North America. These efforts will not only promote plant-based protein consumption but also align with global goals to improve public health, enhance food security, and ensure environmental sustainability. Unlocking the full potential of faba beans will support the transition to more resilient and sustainable food systems, particularly in underutilized markets in North America, including the U.S.

## Author contributions

Madhvi Singh wrote the original draft. All authors contributed to the review and editing process and approved the final version of the manuscript for publication. Dr. Carneiro and Dr. O'Keefe provided funding support for this work.

## Declaration of competing interest

The authors declare the following financial interests/personal relationships which may be considered as potential competing interests:Sean O′ Keefe and Renata Carneiro reports financial support was provided by 10.13039/100007263Virginia Polytechnic Institute and State University. Sean O'Keefe and Renata Carneiro reports a relationship with Virginia Polytechnic Institute and State University that includes: employment. If there are other authors, they declare that they have no known competing financial interests or personal relationships that could have appeared to influence the work reported in this paper.

## Data Availability

No data was used for the research described in the article.

## References

[bib1] Abete I., Romaguera D., Vieira A.R., Lopez De Munain A., Norat T. (2014). Association between total, processed, red and white meat consumption and all-cause, CVD and IHD mortality: a meta-analysis of cohort studies. Br. J. Nutr..

[bib2] Adamidou S., Nengas I., Grigorakis K., Nikolopoulou D., Jauncey K. (2011). Chemical composition and antinutritional factors of field peas (pisum sativum), chickpeas (Cicer arietinum), and faba beans (Vicia faba) as affected by extrusion preconditioning and drying temperatures. Cereal Chem..

[bib3] Adamidou S., Nengas I., Grigorakis K., Nikolopoulou D., Jauncey K. (2011). Chemical composition and antinutritional factors of field peas (Pisum sativum), Chickpeas (Cicer arietinum), and faba beans (Vicia faba) as affected by extrusion preconditioning and drying temperatures. Cereal Chem..

[bib4] Ali H.K., Shakir K.A. (2023). Vicine and convicine levels in dry and fresh beans during the growth stages and the effect of enzymatic treatment and processing conditions on their removal. Iraqi J. Agric. Sci..

[bib5] Alonso R., Aguirre A., Marzo F. (2000). Effects of extrusion and traditional processing methods on antinutrients and in vitro digestibility of protein and starch in faba and kidney beans. Food Chem..

[bib6] Arbid M.S.S., Marquardt R.R. (1985). Hydrolysis of the toxic constituents (vicine and convicine) in fababean (Vicia faba L.) food preparations following treatment with β-glucosidase. J. Sci. Food Agric..

[bib7] Augustin M.A., Cole M.B. (2022). Towards a sustainable food system by design using faba bean protein as an example. Trends Food Sci. Technol..

[bib8] Aune D. (2011). Dietary fibre, whole grains, and risk of colorectal cancer: systematic review and dose-response meta-analysis of prospective studies. Br. Med. J..

[bib9] Badjona A., Bradshaw R., Millman C., Howarth M., Dubey B. (2023). Faba bean flavor effects from processing to consumer Acceptability. Foods 2023.

[bib10] Beretta A., Manuelli M., Cena H. (2023). Favism: clinical features at different ages. Nutrients.

[bib11] Bjerg B., Eggum B.O., Jacobsen I., Olsen O., Sørensen H. (1984). Protein quality in relation to antinutritional constituents in faba beans (Vicia faba L.). The effects of vicine, convicine and dopa added to a standard diet and fed to rats. Z. für Tierphysiol. Tierernaehrung Futtermittelkd..

[bib12] Björnsdotter E. (2021). VC1 catalyzes a key step in the biosynthesis of vicine in faba bean. Nat. Plants.

[bib13] Çalışkantürk Karataş S., Günay D., Sayar S. (2017). In vitro evaluation of whole faba bean and its seed coat as a potential source of functional food components. Food Chem..

[bib14] Cappellini M., Fiorelli G. (2008). Glucose-6-phosphate dehydrogenase deficiency. Lancet.

[bib15] Cardador-Martínez A. (2012). Effect of roasting and boiling on the content of vicine, convicine and L-3,4-dihydroxyphenylalanine in Vicia faba L. J. Food Qual..

[bib16] Chen L., Gupta A., Liu H. (2017). Antinutritional factors in legumes: processing techniques and impacts on health and nutrition. Nutr. Rev..

[bib17] Cheynier V. (2005). Polyphenols in foods are more complex than often thought. Am. J. Clin. Nutr..

[bib18] Clemente A., Olias R. (2017). Beneficial effects of legumes in gut health. Curr. Opin. Food Sci..

[bib19] Coda R. (2015). Effect of air classification and fermentation by Lactobacillus plantarum VTT E-133328 on faba bean (Vicia faba L.) flour nutritional properties. Int. J. Food Microbiol..

[bib20] Collier H.B. (1976). The estimation of vicine in fababeans by an ultraviolet spectrophotometric method. Can. Inst. Food Sci. Technol. J..

[bib21] Davis S., Kim Y. (2021). Effects of glycoside metabolites on cellular health and longevity. J. Cell. Biochem..

[bib22] Dewar J., Taylor J.R.N., Berjak P. (1997). Determination of improved steeping conditions for Sorghum Malting. J. Cereal. Sci..

[bib24] Diez de Fuentes (2016).

[bib25] El-Hady E.A Abd, Habiba R.A. (2003). Effect of soaking and extrusion conditions on antinutrients and protein digestibility of legume seeds. LWT - Food Sci. Technol. (Lebensmittel-Wissenschaft -Technol.).

[bib26] Frenda A.S. (2013). The critical period of weed control in faba bean and Chickpea in Mediterranean areas. Weed Sci..

[bib27] Getachew F., Vandenberg A., Smits J. (2018). A practical toxicity bioassay for vicine and convicine levels in faba bean (Vicia faba). J. Sci. Food Agric..

[bib28] Gilani G.S., Xiao C.W., Cockell K.A. (2012). Impact of antinutritional factors in food proteins on the digestibility of protein and the bioavailability of amino acids and on protein quality. Br. J. Nutr..

[bib29] Giménez M.A., Drago S.R., De Greef D., Gonzalez R.J., Lobo M.O., Samman N.C. (2012). Rheological, functional and nutritional properties of wheat/broad bean (Vicia faba) flour blends for pasta formulation. Food Chem..

[bib30] Goyoaga C. (2008). Content and distribution of vicine, convicine and l-DOPA during germination and seedling growth of two Vicia faba L. varieties. Eur. Food Res. Technol..

[bib31] Gu B.J., Masli M.D.P., Ganjyal G.M. (2020). Whole faba bean flour exhibits unique expansion characteristics relative to the whole flours of lima, pinto, and red kidney beans during extrusion. J. Food Sci..

[bib32] Haciseferoǧullari H., Gezer I., Bahtiyarca Y., Mengeş H.O. (2003). Determination of some chemical and physical properties of Sakiz faba bean (Vicia faba L. Var. major). J. Food Eng..

[bib33] Hansen L.M., Lorentsen L., Boelt B. (2008). How to reduce the incidence of black bean aphids (Aphis fabae Scop.) attacking organic growing field beans (Vicia faba L.) by growing partially resistant bean varieties and by intercropping field beans with cereals. Acta Agric Scand B Soil Plant Sci.

[bib34] Hardarson G., Danso S.K.A., Zapata F., Reichardt K. (1991). Measurements of nitrogen fixation in fababean at different N fertilizer rates using the 15N isotope dilution and ‘A-value’ methods. Plant Soil.

[bib35] Hutchins A.M., Winham D.M., Thompson S.V. (2012). Phaseolus beans: impact on glycaemic response and chronic disease risk in human subjects. Br. J. Nutr..

[bib36] Jakubczyk A., Karaś M., Złotek U., Szymanowska U., Baraniak B., Bochnak J. (2019). Peptides obtained from fermented faba bean seeds (Vicia faba) as potential inhibitors of an enzyme involved in the pathogenesis of metabolic syndrome. Lebensm. Wiss. Technol..

[bib37] Jamalian Jalal (1999). Removal of favism-inducing factors vicine and convicine and the associated effects on the protein content and digestibility of fababeans (Vicia faba L). J. Sci. Food Agric..

[bib38] Jamalian J., Ghorbani M. (2005). Extraction of favism-inducing agents from whole seeds of faba bean (Vicia faba L var major). J. Sci. Food Agric..

[bib39] Jensen E.S., Peoples M.B., Hauggaard-Nielsen H. (2010). Faba bean in cropping systems. Field Crops Res..

[bib40] Jezierny D., Mosenthin R., Bauer E. (2010). The use of grain legumes as a protein source in pig nutrition: a review. Anim. Feed Sci. Technol..

[bib41] Johns P.W., Hertzler S.R. (2021). Substantial depletion of vicine, Levodopa, and Tyramine in a fava bean protein-based nutritional product. Int J Food Sci.

[bib42] Jones P. (2018). Structural analysis of pyrimidine glycosides in selected legumes. J. Agric. Food Chem..

[bib43] Kader Z.M.A. (1995). Study of some factors affecting water absorption by faba beans during soaking. Food Chem..

[bib44] Karsma A. (2015). Bioprocessing with enzymes and lactic acid bacteria for production of new functional faba bean ingredients: master's thesis. https://cris.vtt.fi/en/publications/bioprocessing-with-enzymes-and-lactic-acid-bacteria-for-productio.

[bib45] Khamassi K. (2013). A baseline study of vicine-convicine levels in faba bean (Vicia faba L.) germplasm. Plant Genet. Resour. Charact. Util..

[bib46] Khazaei H. (2017). Development and validation of a robust, breeder-friendly molecular marker for the vc - locus in faba bean. Mol. Breed..

[bib47] Khazaei H. (2019). Eliminating vicine and convicine, the main anti-nutritional factors restricting faba bean usage. Trends Food Sci. Technol..

[bib48] Khazaei H. (2021). Recent advances in faba bean genetic and genomic tools for crop improvement. Legume Science.

[bib49] Kim S.I., Hoehn E., Eskin N.A.M., Ismail F. (1982). A simple and rapid colorimetric method for determination of vicine and convicine. J. Agric. Food Chem..

[bib50] Kumar A., Nidhi, Prasad N., Sinha S.K. (2015). Nutritional and antinutritional attributes of faba bean (Vicia faba L.) germplasms growing in Bihar, India. Physiol. Mol. Biol. Plants.

[bib51] Lee J. (2019). G6PD deficiency and oxidative stress: implications for the understanding of favism. Med. Hypotheses.

[bib52] Lee C., Nguyen M. (2019). Developmental patterns of vicine and convicine accumulation in faba beans. J. Plant Physiol..

[bib53] Liu C., Pei R., Heinonen M. (2022). Faba bean protein: a promising plant-based emulsifier for improving physical and oxidative stabilities of oil-in-water emulsions. Food Chem..

[bib54] Liu R. (2025). A special short-wing petal faba genome and genetic dissection of floral and yield-related traits accelerate breeding and improvement of faba bean. Genome Biol..

[bib55] Liu R. (2025). A special short-wing petal faba genome and genetic dissection of floral and yield-related traits accelerate breeding and improvement of faba bean. Genome Biol..

[bib56] Luo Y.W., Xie W.H. (2013). Effect of different processing methods on certain antinutritional factors and protein digestibility in green and white faba bean (Vicia faba L.). CyTA - J. Food.

[bib57] Luo Y., Xie W., Cui Q. (2010). Effects of phytases and dehulling treatments on in vitro iron and zinc bioavailability in faba bean (Vicia faba L.) flour and legume fractions. J. Food Sci..

[bib58] Maalouf F. (2019). Breeding and genomics status in faba bean (Vicia faba). Plant Breed..

[bib59] Maqbool A., Shafiq S., Lake L. (2010). Radiant frost tolerance in pulse crops-a review. Euphytica.

[bib60] Martineau-Côté D., Achouri A., Karboune S., L'Hocine L. (2022). Faba bean: an untapped source of quality plant proteins and bioactives. Nutrients.

[bib61] Martineau-Côté D., Achouri A., Karboune S., L'Hocine L. (2022). Faba bean: an untapped source of quality plant proteins and bioactives. Nutrients.

[bib62] Martinez A., Rodriguez C. (2022). Biotechnological approaches to antinutritional factors in legumes. Biotechnol. Adv..

[bib63] Martini S., Conte A., Cattivelli A., Tagliazucchi D. (2021). Domestic cooking methods affect the stability and bioaccessibility of dark purple eggplant (Solanum melongena) phenolic compounds. Food Chem..

[bib64] Mayer Labba I.C., Frøkiær H., Sandberg A.S. (2021). Nutritional and antinutritional composition of fava bean (Vicia faba L., var. minor) cultivars. Food Res. Int..

[bib65] McKay A.M. (1992). Hydrolysis of vicine and convicine from fababeans by microbial beta-glucosidase enzymes. J. Appl. Bacteriol..

[bib66] Millar K.A., Gallagher E., Burke R., McCarthy S., Barry-Ryan C. (2019). Proximate composition and anti-nutritional factors of fava-bean (Vicia faba), green-pea and yellow-pea (Pisum sativum) flour. J. Food Compos. Anal..

[bib67] Mudryj A.N., Yu N., Aukema H.M. (2014). Nutritional and health benefits of pulses. Appl. Physiol. Nutr. Metabol..

[bib68] Muhu-Din Ahmed H.G., Naeem M., Faisal A., Fatima N., Tariq S., Owais M. (2023). Enriching the content of proteins and essential amino acids in legumes. Legumes Biofortification.

[bib69] Oatway L., Vasanthan T., Helm J.H. (2001). Phytic acid. Food Rev. Int..

[bib70] O'Sullivan D.M., Angra D. (2016). Advances in faba bean genetics and genomics. Front. Genet..

[bib71] Pampana S., Masoni A., Arduini I. (2016). Response of cool-season grain legumes to waterlogging at flowering. Can. J. Plant Sci..

[bib72] Paramashivam S.K., Balasubramaniam S., Dhiraviam K.N. (2021). Computational exploration of vicine - an alkaloid glycoside mediated pathological hallmark of adenosine kinase to promote neurological disorder. Metab. Brain Dis..

[bib73] Pitz W.J., Sosulski F.W., Hogge L.R. (1980). Occurrence of vicine and convicine in seeds of some vicia species and other pulses. Can. Inst. Food Sci. Technol. J..

[bib74] Polzonetti V., Passini V., Lucarini N. (2011). Association between ACP(1) genetic polymorphism and favism. Genet. Mol. Res..

[bib75] Pulkkinen M. (2019).

[bib76] Pulkkinen M. (2015). Determination of vicine and convicine from faba bean with an optimized high-performance liquid chromatographic method. Food Res. Int..

[bib77] Pulkkinen M., Coda R., Lampi A.M., Varis J., Katina K., Piironen V. (2019). Possibilities of reducing amounts of vicine and convicine in faba bean suspensions and sourdoughs. Eur. Food Res. Technol..

[bib78] Purves R.W. (2018). Enhancing biological LC-MS analyses using ion mobility spectrometry. Compr. Anal. Chem..

[bib79] Purves R.W., Zhang H., Khazaei H., Vandenberg A. (2017). Rapid analysis of medically relevant compounds in faba bean seeds using FAIMS and mass spectrometry. Int. J. Ion Mobility Spectrom..

[bib80] Purves R.W., Khazaei H., Vandenberg A. (2018). Quantification of vicine and convicine in faba bean seeds using hydrophilic interaction liquid chromatography. Food Chem..

[bib81] Purves R.W., Khazaei H., Vandenberg A. (2018). Toward a high-throughput method for determining vicine and convicine levels in faba bean seeds using flow injection analysis combined with tandem mass spectrometry. Food Chem..

[bib82] Quemener B. (1988). Improvements in the high-pressure liquid chromatographic determination of amino sugars and α-Galactosides in faba bean, lupine, and pea. J. Agric. Food Chem..

[bib83] Rahate K.A., Madhumita M., Dattatraya M. (2020). Nutritional composition and health benefits of faba bean (Vicia faba L.) in human diet. JNB (J. Nutr. Biochem.).

[bib84] Rahate K.A., Madhumita M., Prabhakar P.K. (2021). Nutritional composition, anti-nutritional factors, pretreatments-cum-processing impact and food formulation potential of faba bean (Vicia faba L.): a comprehensive review. Lebensm. Wiss. Technol..

[bib85] Ramsay G., Griffiths D.W. (1996). Accumulation of vicine and convicine in vicia faba and V. Narbonensis. Phytochemistry.

[bib86] Reading N.S. (2016). Favism, the commonest form of severe hemolytic anemia in Palestinian children, varies in severity with three different variants of G6PD deficiency within the same community. Blood Cells Mol. Dis..

[bib87] Rinne M. (2020). Fermentation quality of ensiled crimped faba beans using different additives with special attention to changes in bioactive compounds. Anim. Feed Sci. Technol..

[bib88] Rizzello C.G. (2016). Degradation of vicine, convicine and their aglycones during fermentation of faba bean flour. Sci. Rep..

[bib89] Roland W.S.U., Pouvreau L., Curran J., Van De Velde F., De Kok P.M.T. (2017). Flavor aspects of pulse ingredients. Cereal Chem..

[bib90] Rounsevell M.D.A. (1996).

[bib91] Saldanha do Carmo C. (2020). Is dehulling of peas and faba beans necessary prior to dry fractionation for the production of protein- and starch-rich fractions? Impact on physical properties, chemical composition and techno-functional properties. J. Food Eng..

[bib92] Salvador-Reyes R., Furlan L.C., Martínez-Villaluenga C., Dala-Paula B.M., Clerici M.T.P.S. (2023). From ancient crop to modern superfood: exploring the history, diversity, characteristics, technological applications, and culinary uses of Peruvian fava beans. Food Res. Int..

[bib93] Šarauskis E., Romaneckas K., Jasinskas A., Kimbirauskienė R., Naujokienė V. (2020). Improving energy efficiency and environmental mitigation through tillage management in faba bean production. Energy.

[bib94] Schwediauer P., Hagmüller W., Zollitsch W. (2018). Germination of faba beans (Vicia faba L.) for organic weaning piglets. Organic Agric..

[bib95] Sejr Olsen H., Hinge Andersen J. (1978). The estimation of vicine and convicine in fababeans (Vicia faba L.) and isolated fababean proteins. J. Sci. Food Agric..

[bib96] Sergeant K. (2024). Exploration of the diversity of vicine and convicine derivatives in faba bean (Vicia faba L.) cultivars: insights from LC-MS/MS spectra. Molecules.

[bib97] Setia R., Dai Z., Nickerson M.T., Sopiwnyk E., Malcolmson L., Ai Y. (2019). Impacts of short-term germination on the chemical compositions, technological characteristics and nutritional quality of yellow pea and faba bean flours. Food Res. Int..

[bib98] Sharan S. (2021). Fava bean (Vicia faba L.) for food applications: from seed to ingredient processing and its effect on functional properties, antinutritional factors, flavor, and color. Compr. Rev. Food Sci. Food Saf..

[bib99] Sharma G., Srivastava A., Pharmacology D.P.-, undefined (2011). Phytochemicals of nutraceutical importance: their role in health and diseases. academia.eduG Sharma, AK Srivastava, D PrakashPharmacology, 2011•academia.edu.

[bib100] Singh Jyoti, Kaur Arora Simran (2023). Antinutritional factors in plant based foods. Int. J. Agric. Sci..

[bib101] Singh A.K., Bhardwaj R., Singh I.S. (2014). Assessment of nutritional quality of developed faba bean (Vicia faba L.) lines. Journal of AgriSearch.

[bib102] Singh B., Kaur A., Malik K. (2018). Impact of vicine and convicine content on livestock: health implications and breeding strategies. Anim. Nutr..

[bib103] C. G. Sintes, S. Bernal, E. Rojas, P. L. Alegretti, M. N. F. Orella, and A. J. B. Roig, “Favism in the Elderly,” Arch. Clin. Med. Case Rep., vol. 6, no. 4, pp. 582–585, Accessed: November. 24, 2024. [Online]. Available: http://www.fotunejournals.com/favism-in-the-elderly.html.

[bib104] Sixdenier G., Cassecuelle F., Guillaumin L., Duc G. (1996). Rapid spectrophotometric method for reduction of vicine and convicine in faba bean seed. https://www.cabidigitallibrary.org/doi/full/10.5555/19981603438.

[bib105] Sköld M.B., Svendsen R.P., Pedersen E.B. (2017). [Favism after ingestion of fava beans in a three-year-old child with glucose-6-phosphate dehydrogenase deficiency]. Ugeskr Laeger.

[bib106] Smith A., Johnson S., Wilson L. (2020). Identification and quantification of pyrimidine glycosides in faba beans. Phytochemistry.

[bib107] Sosulski F.W., McCURDY A. (1987). Functionality of flours, protein fractions and isolates from field peas and faba bean. J. Food Sci..

[bib108] Sozer N., Melama L., Silbir S., Rizzello C.G., Flander L., Poutanen K. (2019). Lactic acid fermentation as a pre-treatment process for faba bean flour and its effect on textural, structural and nutritional properties of protein-enriched gluten-free faba bean breads. Foods.

[bib109] Tanno K.I., Willcox G. (2006). The origins of cultivation of Cicer arietinum L. and Vicia faba L.: early finds from Tell el-Kerkh, north-west Syria, late 10th millennium B.P. Veg. Hist. Archaeobotany.

[bib110] Tiwari B.K., Singh N. (2023).

[bib111] van Barneveld R.J. (1999). Understanding the nutritional chemistry of lupin (Lupinus spp.) seed to improve livestock production efficiency. Nutr. Res. Rev..

[bib112] Verni M. (2017). Exploring the microbiota of faba bean: functional characterization of lactic acid bacteria. Front. Microbiol..

[bib113] Vidal-Valverde C., Frias J., Sotomayor C., Diaz-Pollan C., Fernandez M., Urbano G. (1998). Nutrients and antinutritional factors in faba beans as affected by processing. Zeitschrift fur Lebensmittel -Untersuchung und -Forschung.

[bib114] Vioque J., Alaiz M., Girón-Calle J. (2012). Nutritional and functional properties of Vicia faba protein isolates and related fractions. Food Chem..

[bib115] Wang Y.W.L. (2015). Effect of processing on phenolic content and antioxidant activity of four commonly consumed pulses in China. J. Hortic..

[bib116] Watson R., Holmes M., Holmes M. (2020). Marker-assisted selection in crop breeding: progress and prospects for faba beans. Genet. Resour. Crop Evol..

[bib117] Webb A. (2016). A SNP-based consensus genetic map for synteny-based trait targeting in faba bean (Vicia faba L.). Plant Biotechnol. J..

[bib118] xiu Xiao J., an Zhu Y., lian Bai W., yang Liu Z., Tang L., Zheng Y. (2021). Yield performance and optimal nitrogen and phosphorus application rates in wheat and faba bean intercropping. J. Integr. Agric..

[bib119] Yang J., Liu G., Zeng H., Chen L. (2018). Effects of high pressure homogenization on faba bean protein aggregation in relation to solubility and interfacial properties. Food Hydrocoll..

[bib120] Yang J., Liu G., Zeng H., Chen L. (2018). Effects of high pressure homogenization on faba bean protein aggregation in relation to solubility and interfacial properties. Food Hydrocoll..

[bib121] Zanotto S., Khazaei H., Elessawy F.M., Vandenberg A., Purves R.W. (2020). Do faba bean genotypes carrying different zero-tannin genes (zt1 and zt2) differ in phenolic profiles?. J. Agric. Food Chem..

[bib122] Faostat 2021 (2021).

[bib123] Global legumes market Worth USD 75.8 billion by 2025: hexa research. https://www.prnewswire.com/news-releases/global-legumes-market-worth-usd-75-8-billion-by-2025-hexa-research-300817529.html.

[bib124] USDA FoodData central https://fdc.nal.usda.gov/.

[bib125] World Food and Agriculture (2024).

